# Telomere length and survival in primary cutaneous melanoma patients

**DOI:** 10.1038/s41598-018-29322-9

**Published:** 2018-07-19

**Authors:** Sivaramakrishna Rachakonda, Nalini Srinivas, Seyed Hamidreza Mahmoudpour, Zaida Garcia-Casado, Celia Requena, Victor Traves, Virtudes Soriano, Maurizio Cardelli, Dace Pjanova, Anders Molven, Nelleke Gruis, Eduardo Nagore, Rajiv Kumar

**Affiliations:** 1 Division of Molecular Genetic Epidemiology, German Cancer Research Center, Heidelberg, Germany; 2 Institute of Medical Biostatistics, University Medical Center of Johannes Gutenberg, University of Mainz, Mainz, Germany; 3 Labortory of Molecular Biology, Instituto Valenciano de Oncologia, Valencia, Spain; 4 Department of Dermatology, Instituto Valenciano de Oncologia, Valencia, Spain; 5 Department of Pathology, Instituto Valenciano de Oncologia, Valencia, Spain; 6 Department of Medical Oncology, Instituto Valenciano de Oncologia, Valencia, Spain; 7 Advanced Technology Center for Aging Research, Italian National Research Center on Aging (INRCA), Ancona, Italy; 8 Latvian Biomedical Research and Study Centre, Riga, Latvia; 9 Department of Clinical Medicine, Gade Laboratory of Pathology, Haukeland University Hospital, Bergen, Norway; 10 Department of Pathology, Haukeland University Hospital, Bergen, Norway; 11 Department of Dermatology, Leiden University Medical Center, Leiden, The Netherlands; 12 German Consortium for Translational Research, German Cancer Research Center, Heidelberg, Germany

## Abstract

Telomere repeats at chromosomal ends, critical to genomic integrity, undergo age-dependent attrition. Telomere length, a polygenic trait, has been associated with risk of several disorders including cancers. In contrast to association of long telomeres with increased risk of several cancers, including melanoma, emerging reports suggest that short telomeres predict poor survival in patients with different cancers. In this study based on 1019 stage I and II cutaneous melanoma patients, we show an association between the patients with short telomeres and poor melanoma-specific survival (HR 2.05, 95% CI 1.33–3.16) compared to patients with long telomeres. Due to inverse correlation between age and telomere length (r -0.19, P < 0.0001), we stratified the patients into quantiles based on age at diagnosis and also carried out age-matched analysis. The effect of short telomeres on survival was determined by using multivariate Cox regression that included composite genetic risk score computed from genotyping of the patients for telomere-length associated polymorphisms. The effect of decreased telomere length on poor melanoma-specific survival was particularly strong in patients within the age quantile below 30 years (HR 3.82, 95% CI 1.10–13.30) and between 30–40 years (HR 2.69, 95% CI 1.03–7.03). Our study shows that in contrast to increased melanoma risk associated with increased telomere length, decreased telomere length predicts poor survival in melanoma subgroups.

## Introduction

Cutaneous melanoma with its propensity to metastasize and intrinsic drug resistance remains causal for the majority of skin cancer related deaths^1,2^. Despite increase in the range of treatments, including targeted and immunotherapies, for eliciting stable response in patients with metastatic melanoma, the long term prospects in terms of treatment remain confined to disease management^3^. The search for the factors that can identify patients at early stages at risk of poor survival remains crucial in melanoma. The known markers of poor outcome in melanoma include increased Breslow thickness, presence of tumor ulceration, increased tumor mitotic rate, reduced nevus number and the presence of locoregional or distant metastasis at diagnosis^4–8^. The melanoma genome in general is characterized by one of the highest prevalence of somatic mutations in human cancers and several genetic alterations have been shown to predict outcome in melanoma^9–13^. In particular, the most frequent somatic mutations in cutaneous melanoma like those in the *TERT* promoter and *BRAF/NRAS* reportedly associate with poor disease-free and melanoma-specific survival^9,14^.

Telomere repeats at chromosomal ends, critical to genomic integrity, are maintained through an elaborate network of proteins and pathways^15,16^. In humans, TTAGGG repeats account for telomere length that ranges between 10–15 kb^17^. Inherent limitations of DNA replication and telomerase suppression in most somatic cells through epigenetic reprogramming of the *telomerase reverse transcriptase* (*TERT*) gene, cause telomeres to undergo age-dependent incremental attrition^16,18^. Telomere shortening is further influenced by oxidative damage and replicative stress caused by genetic, epigenetic and environmental factors^15,19^. However, in most cancers enhanced *TERT* transcription, due to various mechanisms, and consequent telomerase rejuvenation, imparts tumor cells an infinite capability to surmount proliferative barrier through telomere stabilization^16,20,21^. Constitutive telomere length, a polygenic trait with high estimated heritability, has hitherto been demonstrated to be associated with nine different loci, with six harboring genes related to telomere homeostasis^22–25^. Epidemiological data, in general, support an association, with varying magnitudes, between constitutive telomere length and various disorders including cancers^26,27^. Different investigations over the years, in contrast, have suggested that short telomeres associate with poor patient survival^26,28,29^.

To test the association between telomere length and melanoma-specific patient survival, we measured telomere length in constitutive DNA from 1019 incident cutaneous melanoma patients with stage I and II disease. We also used telomere length associated polymorphisms to generate composite genetic score for association with the disease outcome. Despite a confounding effect of the age at diagnosis, our results suggest an association between short telomeres and poor patient survival, particularly for melanoma patients in younger age groups.

## Results

### Relative telomere length and melanoma-specific survival

The study included 1019 patients with AJCC stage I and II cutaneous melanoma. Patients with stage 0, III and IV were excluded from the study (Supplementary Fig. 1). The median age at diagnosis was 52 years [IQR: 39–66] with 463 (45.4%) men and 556 (54.6%) women. Median relative telomere length (represented as the ratio of telomere to single copy gene, T/S) in leukocytes, measured successfully in 995 patients, was 1.20 [inter-quartile range, IQR: 0.96–1.48]. The median follow-up period following the disease diagnosis was 103 months [IQR 60.62–155.11]. Of the 1019 patients, 97 (9.5%) had died due to melanoma. The remaining 922 patients (90.5%) were censored either at their last follow-up date (840 patients) or at death from unrelated causes (82 patients; Supplementary Table S1).

The patients included in the study were further genotyped for rs1317082, rs7726159 and rs6060627 polymorphisms that have been shown to associate with telomere length in genome wide association studies^30^. Since the risk prediction varied with individual polymorphisms, a weighted genetic score was computed based on genotype data **(**Supplementary Table S2**)**. The computed weighted genetic risk score due to genotypes with variant alleles for three polymorphisms ranged from −0.34 to 0.39 for rs1317082, −0.61 to 0.00 for rs7726159 and −0.46 to 0.20 for rs6060627 **(**Supplementary Table S2**)**. The composite genetic risk score, computed for each individual as a sum of weighted scores for all the three polymorphisms, ranged from −1.41 to 0.59 (Median: 0.00; IQR: −0.18 to 0.20).

**Figure 1:**
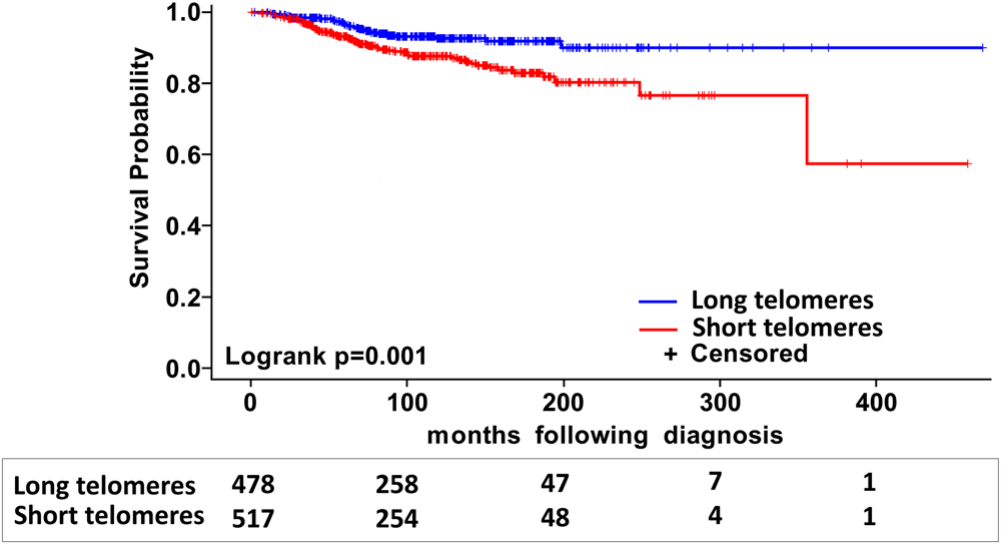
Kaplan-Meier analysis of differences in survival of patients with stage I and II melanoma based on telomere length. Patients were divided into two groups with short (≤1.20) and long (>1.20) telomeres based on median distribution. The numbers of patients at risk in each category, at respective survival intervals, are shown underneath.

In univariate Cox regression analysis with telomere length as a continuous variable, an improved melanoma-specific patient survival was observed with increased telomere length (HR 0.65, 95% CI 0.42–1.00, P 0.05). The telomere data was transformed by taking the natural log of telomere length. On this scale, the effect of each positive unit of ln(telomere length) was estimated to have a HR of 0.46 (95% CI 0.26–0.81) on melanoma-specific survival. The data analysis with telomere length as a dichotomous variable based on median distribution also showed that the patients with short telomeres (≤1.20) were at risk of poor survival (log rank P 0.001; HR 2.05, 95% CI 1.33–3.16, Fig. 1) compared to the patients with long telomeres (>1.20). A similar analysis with telomere length as categorical variable based on quartile distribution showed that the patients in the first quartile compared to those in the fourth quartile (longest telomeres) had the worst survival (HR 2.73, 95% CI 1.47–5.07) followed by patients in the second quartile (HR 1.71, 95% CI 0.88–3.32) and the third quartile (HR 1.42, 95% CI 0.72–2.82).

Other factors that affected patient survival as determined by Kaplan-Meier and univariate Cox regression analyses included age at diagnosis (P < 0.0001), sex (log rank P 0.0006), nevus count (log rank P 0.03), tumor location (log rank P 0.0005), tumor stage (log rank P < 0.0001), Breslow thickness (log rank P < 0.0001) and tumor ulceration (log rank P < 0.0001) **(**Supplementary Table S1**)**. Since tumor stage is defined by Breslow thickness, presence/absence of ulceration and tumor mitotic rate, for multivariate analysis only tumor stage was included in the model.

**Figure 2:**
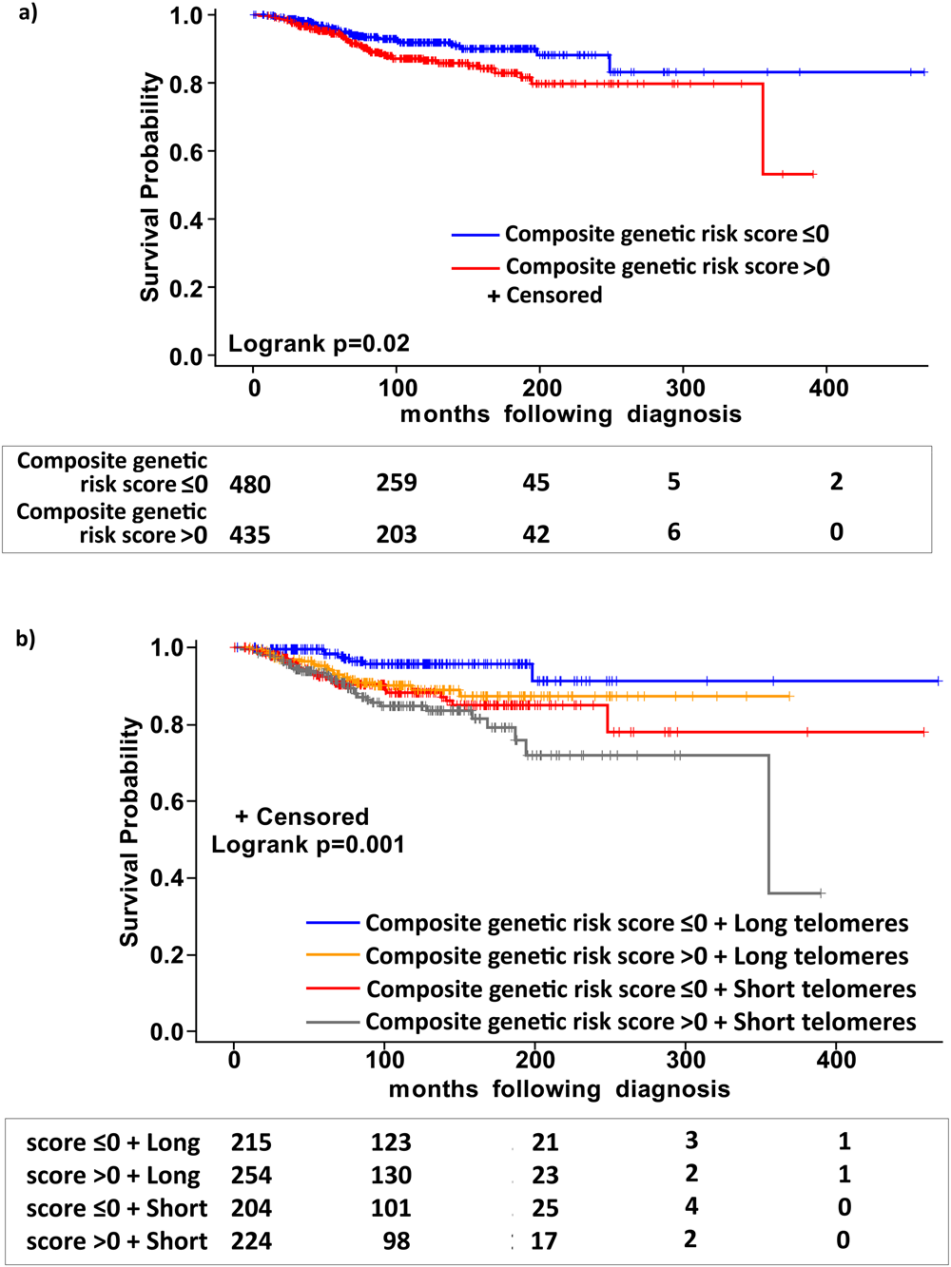
Kaplan-Meier analysis of difference in survival in patients with Stage I and II melanoma; (**a**) after stratification based on the median distribution of composite weighted genetic score; (**b**) after stratification into four sub-groups based on median distributions of composite weighted genetic score and telomere length (i) with composite genetic score ≤0 and long telomeres (ii) with composite genetic score >0 and long telomeres (iii) with composite genetic score ≤0 and short telomeres and (iv) with composite genetic score >0 and short telomeres. The numbers of patients at risk in each category are shown underneath at the respective survival intervals.

As a continuous variable increased composite genetic risk score showed a statistically significant association with poor melanoma-specific survival (HR for per unit of score: 2.94, 95% CI 1.54–5.62). Similarly, a dichotomous model showed that the patients with higher than median composite genetic risk score (>0) were associated with poor melanoma-specific survival compared to patients with lower than median genetic risk score (log rank P 0.02; HR 1.67, 95% CI 1.12–2.49, Fig. 2a). Segregation of patients based on composite risk score and telomere length into 4 categories, showed that the patient group under high-risk category with short telomeres carried the highest risk for mortality (HR 4.28, 95% CI 1.97–9.28) followed by patients in low-risk category with short telomeres (HR 3.13, 95% CI 1.43–6.88), high-risk category with long telomeres (HR 2.51, 95% CI 1.10–5.74) compared to the low-risk category with long telomeres (Log rank P 0.001, Fig. 2b).

### Telomere-length and patient age at diagnosis

We observed a statistically significant inverse correlation (Pearson correlation r −0.19, P < 0.0001) between telomere length and the patient age at diagnosis. A statistically significant interaction (P interaction 0.05) was also observed in the survival analysis that included age, telomere length and age*telomere length interaction term. With inclusion of age in the model, the association between decreased telomere length and survival was no longer statistically significant (HR 1.50, 95% CI 0.96–2.34). None of the other confounders individually affected the association between telomere length and patient survival (data not shown). Therefore, patients were stratified into quantiles based on the age and the effect of telomere length on melanoma-specific survival was measured using the median telomere length for each subgroup separately. The results showed that the patients below 30 years of age at diagnosis (first quantile) with short telomeres were at risk of poor survival (HR 3.91, 95% CI 1.25–12.29) compared to the patients with long telomeres within that sub-group. The corresponding HR for poor survival for patients with short telomeres compared to patients with long telomeres in the second quantile (30–40 years) was 2.89 (95% CI 1.20–6.91). For the third quantile (40–50 years), the corresponding HR was 2.13 (95% CI 1.13–3.99). The association between patients with short telomeres and poor melanoma-specific survival in the fourth quantile (50–60 years) was also statistically significant (HR 1.57, 95% CI 1.00–2.47). For patients in the fifth (60–70 years) and higher quantiles, the similar associations were no t statistically significant **(**Table 1**)**.

**Table 1:** Effect of telomere-length on melanoma-specific survival in different quantiles based on patients age at diagnosis.

		Univariate analysis*			Multivariate analysis^Ɨ^		
Age groups	Telomere length^ǂ^	Number	Dead	HR (95% CI)	Number	Dead	HR (95% CI)^¶^
<30 years	Long (T/S > 1.45)	41	0	ref	36	0	ref
	Short (T/S ≤ 1.45)	48	4	**3.91 (1.25–12.29)**	43	4	**3.82 (1.10–13.30)**
30–40 years	Long (T/S > 1.30)	87	4	ref	73	4	ref
	Short (T/S ≤ 1.30)	91	7	**2.89 (1.20–6.91)**	76	4	**2.69 (1.03–7.03)**
40–50 years	Long (T/S > 1.27)	95	6	ref	80	4	ref
	Short (T/S ≤ 1.27)	99	9	**2.13 (1.13–3.99)**	82	5	1.90 (0.95–3.82)
50–60 years	Long (T/S > 1.10)	98	3	ref	86	2	ref
	Short (T/S ≤ 1.10)	100	6	**1.57 (1.00–2.47)**	90	6	1.34 (0.81–2.23)
60–70 years	Long (T/S > 1.06)	95	10	ref	81	9	ref
	Short (T/S ≤ 1.06)	98	16	1.16 (0.74–1.80)	89	15	0.95 (0.58–1.54)
70–80 years	Long (T/S > 1.02)	57	11	ref	45	9	ref
	Short (T/S ≤ 1.02)	59	11	0.85 (0.47–1.56)	52	9	0.67 (0.35–1.28)
>80 years	Long (T/S > 0.90)	11	3	ref	9	3	ref
	Short (T/S ≤ 0.90)	16	3	0.63 (0.27–1.46)	15	2	0.47 (0.19–1.16)

*Main effects: Telomere length (P 0.02), Age (P < 0.0001); Interaction effect: telomere length*age (P0.05).

^**Ɨ**^Main effects: Telomere length (P 0.03), Age (P 0.0001); Interaction effect: telomere length*age (P0.03).

^**ǂ**^Telomere length was dichotomized at median distribution of T/S ratios within corresponding age groups.

^**¶**^Hazard ratio (HR) in multivariate model was adjusted for composite genetic risk score, gender, number of nevi, tumor location and tumor stage. HR values in bold indicate statistical significance (P < 0.05).

### Multivariate analysis

Multivariate analysis that included age, composite genetic risk score, sex, nevus number, tumor localization, and tumor stage showed that the association of patients with short telomeres (compared to patients with long telomeres) with decreased melanoma-specific survival was not statistically significant (HR 1.17, 95% CI 0.71–1.94, P 0.53; Table 2). A similar result was obtained using telomere length as a continuous variable (HR 0.95, 95% CI 0.67–1.36, P 0.79) and after transformation into log-scale, the HR for the effect of long telomeres on survival was 0.92 (95% CI 0.52–1.62, P 0.76). However, the increased composite genetic risk-score, in the multivariate model, was a statistically significant independent prognostic factor for poor survival (HR 2.75, 95% CI 1.34–5.62). Additionally, the patients with nevus count >50 showed better survival (HR 0.54, 95% CI 0.21–1.38, P 0.20) than those with nevus count <50 (Table 2). Further, multivariate analysis using telomere length and composite genetic risk score as a combined variable showed that the patients under high-risk category with both short and long telomeres showed an increased mortality compared to patients in low-risk category with long telomeres **(**Table 3**)**. Similarly, a multivariate model that included all confounders showed that the patients with short telomeres in the age group below 30 years (first quantile) were at statistically significant risk of poor survival (HR 3.82, 95% CI 1.10–13.30) compared to patients with long telomeres within the same group. A similar statistically significant association between patients with short telomeres and poor melanoma-specific survival was also observed in the age group 30–40 years (HR 2.69, 95% CI 1.03–7.03). The effect of telomere length on patient survival diminished in subsequent quantiles **(**Table 1**)**.

**Table 2:** Multivariate Cox analysis of the effect of telomere-length on melanoma-specific survival.

		N	Dead	HR (95% CI)	P
Telomere length	Long (ratio >1.20)	405	25	ref	
	Short (ratio ≤1.20)	452	51	1.17 (0.71–1.94)	0.53
Composite genetic risk score	*(continuous)*	857	76	**2.75 (1.34–5.62)**	**0.006**
Age at diagnosis	*(continuous)*	857	76	**1.03 (1.02–1.05)**	**0.0001**
Sex	Males	389	43	ref	
	Females	468	33	0.75 (0.45–1.23)	0.25
Nevus count	<50	731	71	ref	
	>50	126	5	0.54 (0.21–1.38)	0.20
Tumor location	Axial	476	50	ref	
	Extremities	322	17	0.57 (0.32–1.02)	0.06
	Acral/Mucosal	59	9	1.00 (0.47–2.12)	1.00
Tumor stage*	1 (1A, 1B)	616	27	ref	
	2 (2A, 2B, 2C)	241	49	**4.05 (2.49–6.60)**	**<0.0001**

*As tumor stage is defined by Breslow thickness, presence/absence of ulceration and tumor mitotic rate, therefore only tumor stage was included in the analysis.

**Table 3:** Multivariate analysis of the effect of telomere length and composite genetic risk score on melanoma-specific survival.

	Composite genetic risk score/Telomere length*	N	Dead	HR (95% CI)^Ɨ^	P
Telomere length	Low risk + long telomeres	208	8	ref	
	Low risk + short telomeres	239	24	1.74 (0.78–3.99)	0.17
	High risk + long telomeres	197	17	**2.41 (1.04–5.61)**	**0.04**
	High risk + short telomeres	213	27	2.19 (0.97–4.91)	0.06

*The composite genetic risk score was dichotomized at median distribution (low risk ≤ 0 and high risk > 0). The telomere length was dichotomized at median distribution of T/S ratios (long > 1.20 and short ≤ 1.20).

^**Ɨ**^Hazard ratio (HR) was adjusted for age at diagnosis, gender, number of nevi, tumor location and tumor stage.

Further we also carried age-matched analysis of the patients with “short” and “long” telomeres. Exact matching of the groups (detailed in methods section) resulted in a subset of 676 patients with 338 in each group with a median age of 52 (Supplementary Fig. 2). Cox regression analysis on the age matched group showed that the patients with short telomeres compared to those with long telomere were at statistically significant risk of poor survival (univariate HR 1.89, 95% CI 1.10–3.25, P 0.02). Multivariate data analysis also showed in age-matched groups, the patients with short telomeres were at the risk of poor survival (HR 1.79, 95% CI 1.01–3.17, P 0.05; Table 4) compared to the patients with long telomeres.

**Table 4:** Multivariate analysis of effect of telomere length on melanoma-specific survival in age-matched groups.

		N	Dead	HR (95% CI)	P
Telomere length	Long (T/S ratio > 1.20)	290	18	ref	
	Short (T/S ratio ≤ 1.20)	308	36	**1.79 (1.01–3.17)**	**0.05**
Composite genetic risk score	*(continuous)*	598	54	**2.89 (1.26–6.59)**	**0.01**
Sex	Males	254	33	ref	
	Females	344	21	**0.49 (0.27–0.87)**	**0.02**
Tumor location	Axial	326	34	ref	
	Extremities	228	12	0.60 (0.30–1.19)	0.14
	Acral/Mucosal	44	8	**2.34 (1.03–5.31)**	**0.04**
Tumor stage*	1 (1A, 1B)	436	22	ref	
	2 (2A, 2B, 2C)	162	32	**3.60 (2.05–6.30)**	**<0.0001**

*As tumor stage is defined by Breslow thickness, presence/absence of ulceration and tumor mitotic rate, therefore only tumor stage was included in the analysis.

## Discussion

In this study based on stage I and II incident melanoma patients, we observed that short telomeres predispose patients to poor melanoma-specific survival, which contrasts with reported association of long telomere with increased risk of melanoma^26,31^. Despite a strong inverse correlation between telomere length and age, we observed that the effect of short compared to long telomeres on poor survival was pronounced in patients below 40 years at diagnosis. We also used three polymorphisms located at the *TERC*, *TERT* and *BCl2L1* loci to generate composite genetic risk score. The polymorphisms that had been previously shown to be associated with telomere length and risk of various cancers through genome wide association studies did not *per se* show any impact on melanoma-specific survival in this study^23,30,32^. However, the use of composite genetic risk score together with telomere length strengthened the prediction of melanoma-specific survival in this study. Our results were further corroborated through use of age-matched analysis, which showed that patients with short telomeres were at a statistically significant poorer risk of survival than the patients with long telomeres.

Increased risk of melanoma due to increased telomere length has been postulated as a paradox where sufficient telomere length allows cells to survive until crisis point through continuous division and consequent acquisition of driver mutations^33^. Multiple alleles that associate with increased telomere length have been shown to increase the risk of various cancers^27,31,34–36^. Many of the variants associated with constitutive telomere length have been shown to functionally increase telomerase levels^37,38^. However, it is the critically short telomeres that trigger events leading to genomic instability through end-to-end chromosomal fusion with ultimate emergence of cells from crisis through increased telomerase levels^39,40^. As shown in this and earlier studies, the shorter telomeres tend to associate with poor survival^41^. In previous studies on breast cancer, multiple myeloma and renal cell carcinoma, short telomeres were shown to be associated with poor overall survival^41–44^. Similarly, decreased telomere length was reported to be independently associated with worst survival in patients with idiopathic pulmonary fibrosis; longer donor leukocyte telomere length associated with increase five year survival in patients receiving hematopoietic cell transplantation for severe aplastic anemia but not for patients with acute leukemia^45–47^. Reduced telomere length in general, in a large prospective study, was shown to be associated with increased risk of infections^48^.

The observed association of short telomeres with poor melanoma-specific survival seems to be in conformity with emerging data^49^. Studies have shown that despite ubiquitous telomerase rejuvenation, a continued shortening of telomeres in tumor cells, particularly in presence of the *TERT* promoter mutations, in the initial stages lead to chromosomal fusions and aneuploidy^21,49^. Longer telomeres afford ample time for continued cell division until telomerase rejuvenation and telomere stabilization, hence the association with increased risk. In contrast, once a patient has developed the disease, it is the shorter telomeres in tumors that would presumably lead to rapid chromosomal fusions and aneuploidy resulting in observed poor outcome^49,50^. Evidence suggests that genetic instability drives tumorigenesis^51^. Distinct types of aneuploidy predict two main hallmarks of cancer, cell proliferation and immune evasion; tumor aneuploidy was reported to correlate inversely with patient survival in clinical trials of immune checkpoint blockade anti-CTLA-4 therapy for metastatic melanoma^52^.

Previously, we have shown that the *TERT* promoter mutations associated with poor survival in patients with primary melanoma and our subsequent data showed shorter telomeres in tumors with than without the *TERT* promoter mutations^9,53–55^. In this study, telomere length was measured in blood cells and not in tumor tissues; therefore, the association of short telomere length with decreased survival cannot be directly explained by an effect in tumor cells. The telomere attrition in blood leukocytes has been considered as a marker of biological age and also associated with immune dysfunctions^56,57^. However, we lacked data on immune competency for the patients included in the study to investigate any such correlation.

In this study, however, we show that in contrast to the association between long telomeres and increased melanoma risk, short telomeres predict poor melanoma-specific survival. Other factors that associated with poor survival, besides age at diagnosis, included sex, tumor stage, and reduced nevus count. While increased nevus number is an established a risk factor in melanoma, an earlier report had also shown that high nevus count confers a favorable outcome in melanoma^5,58^. One report also linked increased nevus number with longer telomeres^59^. With age as a strong confounder, the risk of poor survival due to short telomeres on survival was in particularly pronounced in younger patients. However, it may be pointed out that the telomere length can be affected a number of other factors including chronic diseases^60,61^. The lack of relevant data on those factors remains a weakness of the present study and requires cautious interpretation of the results.

## Methods

### Patient material

The patients included in the present study came from the database maintained at the Instituto Valenciano de Oncologia, a referral skin cancer center for the provinces of Valencia, Alicante and Castellon with a catchment population of approximately 5 million. The database contained a total of 2471 melanoma patients. The number of incident cases recruited between 200 and 2014 was 1712. For this study we included incident cases from which we excluded stage 0 (298 patients), stage III (294 patients), stage IV (31 patients) and 30 patients with melanoma at extra-cutaneous sites. All cases had been staged including lymph node biopsy. Thus, for this study 1059 patients with localized invasive stage I and II melanoma were eligible; however for 40 patients, blood samples were not available. At the end we included 1019 incident cases with histologically confirmed stage I and II melanoma (Supplementary Fig. 1).

Blood samples from the selected patients at the time of diagnosis were collected after informed patient consent between 2000 and 2014 and information remains documented in the departmental database. The patients also provided informed consent to the use of their clinical records in this study. Clinical and pathological data from patients were collected since January 2000 through the review of medical history, personal interview, and clinical examination by expert dermatologists (EN and CR) at the first visit. The available patient information included details about the patient age at diagnosis, sex, nevus count, tumor location, tumor stage, Breslow thickness, tumor ulceration, tumor mitotic rate, histology, lentigines, non-melanoma skin cancer, tumor regression, and smoking **(**Supplementary Table 1**)**.

The study was approved by the IVO Ethics Committee and all methods were performed in accordance with the relevant guidelines and regulations. DNA from blood was extracted using standard kits (Qiagen).

### Relative telomere length measurement

The relative telomere length (TL) was measured in DNA from peripheral blood leucocytes using a monochrome multiplex quantitative real-time PCR (MMqPCR) method that compares telomere repeat copy number (T) to the copy number of a single-copy gene, albumin (S). The relative TL in a given DNA sample was calculated as a corresponding T/S ratio^62,63^. Each MMqPCR optical 384-well reaction plate contained DNA in triplicates from blood leucocytes, standard DNA and non-template controls. The standard DNA was obtained by pooling the leucocyte genomic DNA from 15 healthy individuals (40–50 years of age). A two-fold serial dilution of standard DNA in seven concentrations (30 ng to 0.47 ng) was used in each plate for quantification. The PCR reaction was performed in a 10 μL reaction volume using 2 μL of 5 × HOT FIREPol Probe qPCR Mix Plus with ROX (Solis BioDyne, Tartu, Estonia), 1.5 μM of Syto 9 (Invitrogen, Carlsbad, CA) and 2–5 ng of genomic DNA. Four primers were used in each reaction to amplify telomere repeats (telg at 200 nM and telc at 400 nM) and albumin gene (albugcr2 at 200 nM and albdgcr2 at 400 nM). The real-time PCR experiments were carried out using the sequence detection system (Applied Biosystems Viia-7) using two simultaneous programs to acquire the respective Ct values for telomere repeats and albumin (control) gene. The conditions for amplification of telomere repeats were 95 °C/15 min, 2 cycles of 95 °C/20 sec and 49 °C/1 min, followed by 25 cycles of 85 °C/20 sec with signal acquisition at 59 °C/30 sec. Thermal conditions for albumin gene were 35 cycles of 95 °C/15 sec, 85 °C/30 sec, with signal acquisition at 84 °C/30 sec. The specificity of all amplifications was determined by melting curve analysis done at default settings (95 °C/15 sec, 60 °C/1 min with continuous signal acquisition at 0.05 °C/sec ramping, 95 °C/15 sec). The efficiency of PCR amplification and T and S values for each sample were determined from the respective standard curves of telomere and albumin reactions. The RTL was expressed as the ratio between T/S by taking mean of the triplicate values for each sample. Inter-assay variation and intra-assay variation was determined by duplicating the reference DNA for all the dilutions in all the assays performed. The PCR efficiencies for telomere and albumin gene assay ranged between 96% and 101%. The interassay coefficients of variation of the telomere and albumin gene assays were 3.5% and 2.67%, respectively. The intra-assay coefficients of variations of the telomere and albumin gene assays were 1.04% and 0.55%, respectively.

### Statistical analysis

Differences between the patient groups with short and long telomeres were assessed using chi-square test for categorical variables or two sample t-tests for continuous variables. The differences in telomere length in different sample batches procured at different time points were assessed using one-way ANOVA in generalized linear model. All the statistical tests were two-sided and P < 0.05 was considered significant. The telomere length as T/S ratio was taken as continuous variable (with and without log-transformation) or as categorical variable. The telomere length was dichotomized based on the median distribution, with patients with T/S ratio ≤ 1.20 categorized as the group with ‘short telomeres’ and those with T/S > 1.20 as the group with ‘long’ telomeres. Alternatively, patients were grouped into quartiles based on telomere length with T/S 0.48–0.96 for the first quartile, 0.97–1.19 for the second quartile, 1.20–1.48 for the third quartile and 1.49–4.85 for the fourth quartile (reference). All the other clinical covariates included in the analysis were categorical variables. Patient age was considered as a continuous variable unless otherwise specified.

The Kaplan–Meier method was employed to draw the cumulative survival curves using time period (in months) between the date of diagnosis and the date of cause-specific death (death from melanoma). The patients alive at the end of follow-up period and patients that died from other causes were censored. Death from melanoma was confirmed in most patients by the specialists in charge (E.N. and V.S.), who followed them until the date of death. In a few remaining patients, mainly people living far from the Institute and who spent their last days at home or at the regional hospital, the cause of death was assessed by phone contact with the personnel in charge of the palliative care unit in the area.

Statistical differences between the groups were analyzed by the log-rank test. The association of the independent variables with melanoma specific patient survival was assessed using univariate methods. Hazard ratios (HRs) and 95% confidence intervals (95% CI) were estimated in Cox regression models. For multivariate analysis, the variables that associated statistically significantly (P < 0.05) in univariate analysis, were included in the model. The patient survival time and age were inversely correlated (censored/alive, estimate: −1.08, P > 0.0001; dead, estimate: −1.11, P 0.0004). To measure the telomere length and age interaction on patient survival, a Cox regression model was implemented using telomere length and age as main effects and telomere length*age as interaction effect. For this purpose, patient cohort was divided into 7 quantiles, each representing a 10-year interval based on patient age at diagnosis (1st quantile: <30 years, 2nd quantile: 30–40 years, 3rd quantile: 40–50 years, 4th quantile: 50–60 years, 5th quantile: 60–70 years, 6th quantile: 70–80 years and 7th quantile: >80 years). The telomere length was dichotomized into long and short telomeres based on the median telomere length (T/S ratio) for each quantile. In Cox regression model, telomere length and age were considered as dichotomous and ordinal variable, respectively.

Additionally, to minimize the confounder effect of age in determining the effect of telomere length on patient survival, the two groups of patients with, short (median ≤1.20) and long (>1.20) telomeres were matched for age. Before matching, the number of patients in “short” and “long” telomere group was 517 and 478 with median age 57 years (IQR: 46–68) and 46 years (IQR: 35–61), respectively. To make the groups comparable, an exact matching was carried out using Proc SQL in SAS and the “long” and “short” groups of patients were considered as controls and cases, respectively. The data used for this purpose contained three variables – patient identification, short/long telomeres, and the age at diagnosis. The method of matching was based on (1) for each study case, control cases are matched for age; (2) if a control matched with more than one case, one control was randomly selected; (3) randomly selected desired number of controls for each case. The matching resulted in two identical groups with respect to age, each with 338 patients (Median: 52 years, IQR: 41–65). The method implemented was based on Kawabata *et al*. (2004) Proceedings of the Twenty-Ninth Annual SAS Users Group International Conference. Cary, NC: SAS Institute Inc.

To determine the effect of polymorphisms on patient survival, the genotype data were analyzed under the additive, dominant and recessive models. The polymorphisms were categorized into carriers and non-carriers based on the association of alleles with short telomeres. Further, a weighted genetic risk score was calculated for the three genotypes of each polymorphism from Cox regression analysis. Using homozygous common genotype as the reference, the beta coefficients for the other two genotypes (heterozygote and homozygous variant genotypes) were calculated from Cox regression analysis and used as the corresponding weight. Only patients with complete genotype data for three polymorphisms were included for computing weighted score. The weighted genetic risk score thus calculated for all three SNPs was summed for each individual based on their genotypes to get composite genetic risk score for that individual. The composite genetic risk score was subsequently implemented in the analyses as a continuous variable in the multivariate analyses. To analyze the combined genetic effect and telomere length on survival, patients were categorized into 4 categories as high risk (composite genetic risk score >0) and long telomeres (median >1.20), high risk (composite genetic risk score >0) and short telomeres (median ≤1.20), low risk (composite genetic risk score ≤0) and long telomeres (median >1.20), low risk (composite genetic risk score ≤0) and short telomeres (median ≤1.20). All statistical analyses were carried out using SAS version 9.4 (SAS Institute Inc., Cary, NC).

## Electronic supplementary material

Supplementary Information
