# Author Correction: Telomere length and survival in primary cutaneous melanoma patients

**DOI:** 10.1038/s41598-018-36657-w

**Published:** 2018-12-14

**Authors:** Sivaramakrishna Rachakonda, Nalini Srinivas, Seyed Hamidreza Mahmoudpour, Zaida Garcia-Casado, Celia Requena, Victor Traves, Virtudes Soriano, Maurizio Cardelli, Dace Pjanova, Anders Molven, Nelleke Gruis, Eduardo Nagore, Rajiv Kumar

**Affiliations:** 1 Division of Molecular Genetic Epidemiology, German Cancer Research Center, Heidelberg, Germany; 2 Institute of Medical Biostatistics, University Medical Center of Johannes Gutenberg, University of Mainz, Mainz, Germany; 3 Labortory of Molecular Biology, Instituto Valenciano de Oncologia, Valencia, Spain; 4 Department of Dermatology, Instituto Valenciano de Oncologia, Valencia, Spain; 5 Department of Pathology, Instituto Valenciano de Oncologia, Valencia, Spain; 6 Department of Medical Oncology, Instituto Valenciano de Oncologia, Valencia, Spain; 7 Advanced Technology Center for Aging Research, Italian National Research Center on Aging (INRCA), Ancona, Italy; 8 Latvian Biomedical Research and Study Centre, Riga, Latvia; 9 Department of Clinical Medicine, Gade Laboratory of Pathology, Haukeland University Hospital, Bergen, Norway; 10 Department of Pathology, Haukeland University Hospital, Bergen, Norway; 11 Department of Dermatology, Leiden University Medical Center, Leiden, The Netherlands; 12 German Consortium for Translational Research, German Cancer Research Center, Heidelberg, Germany

Correction to: *Scientific Reports* 10.1038/s41598-018-29322-9, published online 19 July 2018

The original version of this Article contained a typographical error in the spelling of the author Dace Pjanova, which was incorrectly given as Dace Pajnova. This has now been corrected in the PDF and HTML versions of the Article, and in the accompanying Supplementary Information file.

