# Supplementary material for: Telomere length and survival in primary cutaneous melanoma patients
Source: Sci Rep. 2018 Jul 19;8:10947. doi: 10.1038/s41598-018-29322-9 (PMC6053393; doi:10.1038/s41598-018-29322-9)
Supplement: Supplementary file 1 — Supplementary Information [file 41598_2018_29322_MOESM1_ESM.pdf]

Supplementary Information

Telomere length and survival in primary cutaneous melanoma patients

Sivaramakrishna Rachakonda[1], Nalini Srinivas[1], Seyed Hamidreza Mahmoudpour[1,2], Zaida Garcia-Casado[3], Celia Requena[4], Victor Traves[5], Virtudes Soriano[6], Maurizio Cardelli[7], Dace Pjanova[8], Anders Molven[9,10], Nelleke Gruis[11], Eduardo Nagore[4*], Rajiv Kumar[1,12*]

[1]Division of Molecular Genetic Epidemiology, [12]German Consortium for Translational Research, German Cancer Research Center, Heidelberg, Germany

[2]Institute of Medical Biostatistics, University Medical Center of Johannes Gutenberg, University of Mainz, Mainz, Germany

[3]Labortory of Molecular Biology, [4]Department of Dermatology, [5]Department of Pathology, [6]Department of Medical Oncology, Instituto Valenciano de Oncologia, Valencia, Spain

[7]Advanced Technology Center for Aging Research, Italian National Research Center on Aging (INRCA), Ancona, Italy

[8]Latvian Biomedical Research and Study Centre, Riga, Latvia

[9]Department of Clinical Medicine, Gade Laboratory of Pathology, [10]Department of Pathology, Haukeland University Hospital, Bergen, Norway

[11]Department of Dermatology, Leiden University Medical Center, Leiden, The Netherlands

*Equal contribution

***Correspondence to:** Rajiv Kumar, Division of Molecular Genetic Epidemiology, German Cancer Research Center, Im Neuenheumer Feld 580, 69120 Heidelberg, Germany

Email: r.kumar@dkfz.de

Results

Telomere length-associated single nucleotide polymorphisms

The patients included in the study were further genotyped for rs1317082, rs7726159 and rs6060627 polymorphisms that have been shown to associate with telomere length in genome wide association studies[1]. The genotype data were analyzed using additive, dominant and recessive models. The genotypes *per se* did not associate with patient survival (**Supplementary Table S2**). For the rs1317082 A>G polymorphism within the *TERC* locus, the variant G allele associated with a decrease in telomere length by 77 nucleotides[1]. The variant A-allele for the rs7726159 C>A polymorphism within the *TERT* locus has been reported to be associated with an increase in telomere length by 73 nucleotides[1]. Similarly, for the rs6060627 C>T polymorphism at the *BCL2L1* locus, the variant T allele has been shown to be associated with an increase in telomere-length by 36 nucleotides[1].

**Supplementary Table S1.** Clinical-pathological parameters of cutaneous melanoma patients						
		**Total Number**	**Disease-specific survival**			
			**Dead (%)**	**HR (95% CI)**	**P**	**Log rank P**
All categories		1019	97 (9.5)			
Telomere length	Long (T/S ratio>1.20)	478	30 (6.2)	ref		0.001
	Short (T/S ratio≤1.20)	517	63 (12.2)	**2.05 (1.33-3.16)**	0.001	
	Data missing	24	4			
Age	*(Continuous)*	1019	97	**1.04 (1.03-1.06)**	**<0.0001**	-
Sex	Male	463	57 (12.3)	ref	0.0008	0.0006
	Female	556	40 (7.2)	**0.50 (0.33-0.75**)		
Nevus count	<50	832	79 (9.5)	ref	0.03	0.03
	>50	144	7 (4.9)	**0.43 (0.20-0.94)**		
	Data missing	43	11 (25.6)			
Tumor location	Axial (Head&neck /Trunk)	564	65 (11.5)	ref		0.0005
	Extremities (Lower/Upper)	385	21	**0.44 (0.27-0.71)**	0.001	
	Acral /Mucosal	70	11	1.46 (0.77-2.78)	0.24	
Tumor stage	1 (1A, 1B)	737	34 (4.6)	ref	<0.0001	<0.0001
	2 (2A, 2B, 2C)	282	63 (22.3)	**5.39 (3.54-8.22)**		
Breslow thickness	≤2mm	792	46	ref	<0.0001	<0.0001
	>2mm	221	51	**4.75 (3.18-7.09)**		
	missing	6				
Tumor ulceration	No	830	52 (6.3)	ref	<0.0001	<0.0001
	Yes	163	41 (25.2)	**4.95 (3.28-7.48)**		
	Data missing	26	4 (15.4)			
Tumor mitotic rate		707	60 (8.5)	**1.14 (1.09-1.19)**	<0.0001	
	Data missing	312	37 (11.9)			
Histology	LMM	57	4 (7.0)	1.18 (0.43-3.29)	0.75	<0.0001
	SSM	707	47 (6.6)	ref		
	NM	152	30 (19.7)	**3.16 (1.99-5.00)**	<0.0001	
	ALM	35	5 (14.3)	2.41 (0.96-6.06)	0.06	
	others	68	11 (16.2)	**2.79 (1.45-5.39)**	0.002	

Lentigines	No	120	10 (8.3)	ref		0.63
	Yes	852	78 (9.2)	1.18 (0.61-2.27)	0.63	
	Data missing	47	9 (19.1)			
Non-melanoma skin cancer	No	832	80 (9.6)	ref		
	Yes	165	12 (7.3)	0.73 (0.40-1.35)	0.32	0.32
	Data missing	22	5 (22.7)			
Tumor regression	No	777	74 (9.5)	ref		0.90
	50% of tumor	69	4 (5.8)	0.79 (0.29-2.16)	0.64	
	>50% of tumor	14	1 (7.1)	1.04 (0.14-7.48)	0.97	
	Data missing	159	18 (11.3)			
Smoking	No	533	46 (8.6)	ref		0.84
	Yes	418	35 (8.4)	1.05 (0.67-1.63)	0.84	
	Data missing	68	16 (23.5)			

Supplementary Table S2. Effect of telomere-length associated polymorphisms on melanoma-specific survival

**Effect**	Genotype	Number	Dead	**HR (95% CI)**	P-value	Weighted genetic risk score
rs1317082	*AA*	650	56	ref		0
	AG	293	36	1.49 (0.98-2.27)	0.06	0.39
	GG	45	3	0.71 (0.22-2.26)	0.56	-0.34
	Dominant					
	AA	650	56	ref		
	AG+GG	338	39	1.37 (0.91-2.07)	0.13	
	Recessive					
	GG	45	3	ref		
	AA+AG	943	92	1.63 (0.52-5.14)	0.41	
rs7726159	*CC*	369	39	ref		0
	CA	426	41	0.89 (0.57-1.38)	0.60	-0.12
	AA	163	10	0.54 (0.27-1.09)	0.08	-0.61
	Dominant					
	CC	369	39	ref		
	CA+AA	589	51	0.79 (0.52-1.20)	0.27	
	Recessive					
	AA	163	10	ref		
	CC+CA	795	80	1.74 (0.90-3.35)	0.10	
rs6060627	*CC*	397	36	ref		0
	CT	434	48	1.22 (0.79-1.88)	0.37	0.20
	TT	125	7	0.63 (0.28-1.42)	0.27	-0.46
	Dominant					
	CC	397	36	ref		
	CT+TT	559	55	1.09 (0.72-1.66)	0.69	
	Recessive					
	TT	125	7	ref		
	CC+CT	831	84	1.76 (0.82-3.81)	0.15	

1 Pooley, K. A. *et al.* A genome-wide association scan (GWAS) for mean telomere length within the COGS project: identified loci show little association with hormone-related cancer risk. *Human molecular genetics* **22**, 5056-5064, doi:10.1093/hmg/ddt355 (2013).

Legends to Supplementary Figures

Supplementary Figure S1: Flow chart showing the selection of patients for investigation of the effect of telomere length on melanoma-specific survival. Only incident patients with stage I and II melanoma were included in the study.

Supplementary Figure S2: Descriptive statistics of age-matched groups of melanoma patients with long and short telomeres. The exact matching of the patients was carried using Proc SQL in SAS and the patients with long telomeres were considered as controls and the patients with short telomeres as cases.
